# Pathology of three ALS patients with *FUS* variants, including one likely benign Q23L variant lacking FUS inclusions

**DOI:** 10.1093/hmg/ddaf119

**Published:** 2025-07-15

**Authors:** Erica Stenvall, Kornelia Åman Grönlund, Zdenek Rohan, Per Zetterström, Angelica Nordin, Karin Forsberg

**Affiliations:** Department of Medical Biosciences, Umeå University, Universitetstorget 4, SE-901 87 Umeå, Umeå, Sweden; Department of Clinical Sciences, Neurosciences, Umeå University, Universitetstorget 4, SE-901 87 Umeå, Umeå, Sweden; Department of Medical Biosciences, Umeå University, Universitetstorget 4, SE-901 87 Umeå, Umeå, Sweden; Department of Medical Biosciences, Umeå University, Universitetstorget 4, SE-901 87 Umeå, Umeå, Sweden; Department of Medical Biosciences, Umeå University, Universitetstorget 4, SE-901 87 Umeå, Umeå, Sweden; Department of Clinical Sciences, Neurosciences, Umeå University, Universitetstorget 4, SE-901 87 Umeå, Umeå, Sweden

**Keywords:** amyotrophic lateral sclerosis, Neuropathology, Fused in sarcoma, de novo, genetics

## Abstract

Fused in sarcoma (FUS) is an RNA-binding protein implicated in juvenile amyotrophic lateral sclerosis (ALS). Mutations in the *FUS* gene, particularly those affecting the nuclear localization signal (NLS), impair nuclear import and lead to cytoplasmic accumulation of FUS inclusions in motor neurons. However, the pathological and clinical significance of *FUS* variants outside the NLS remains less understood. Here, we describe clinical and histopathological findings from three ALS patients carrying *FUS* variants: two with NLS-region variants (R495X and P525L), and one with a variant in the N-terminal region outside the NLS (Q23L). The patients carrying NLS variants presented with aggressive, juvenile-onset spinal and bulbar ALS, characterized primarily by lower motor neuron involvement and rapid disease progression. In contrast, the Q23L patient exhibited a slowly progressive disease course, with predominantly upper motor neuron signs. Neuropathological analysis revealed cytoplasmic FUS inclusions in motor neurons of patients with NLS variants, consistent with typical FUS pathology. In contrast, the Q23L patient lacked FUS inclusions and instead displayed pTDP-43 pathology in the hippocampus, neocortex (including the motor cortex), nucleus olivaris, lentiform nucleus, striatum, and some lower motor neurons. Taken together, these results suggest that Q23L is most likely a benign variant. As antisense oligonucleotides (ASOs) targeting *FUS* are currently being explored in clinical trials, further neuropathological investigations are needed to determine whether ASO-mediated *FUS* silencing would be effective for patients carrying *FUS* variants outside the NLS region.

## Graphical Abstract

**Graphical Abstract:**
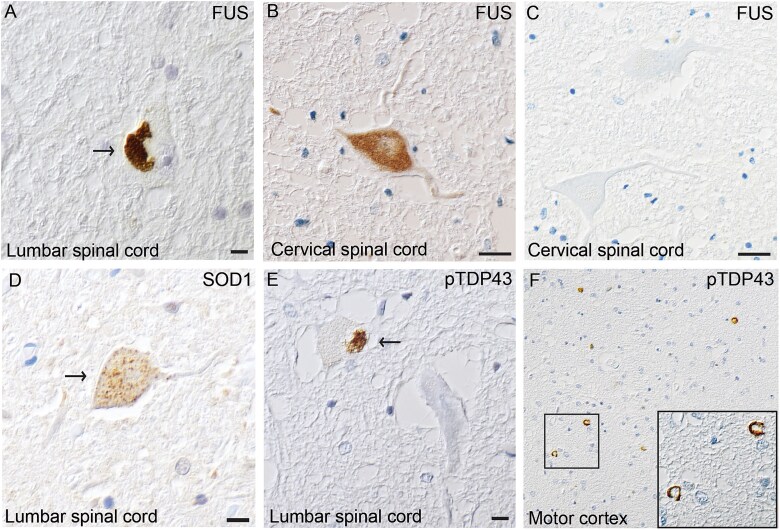

## Introduction

Amyotrophic lateral sclerosis (ALS) is a fatal neurodegenerative disease that primarily affects motor neurons of the brain, brainstem and spinal cord, leading to progressive muscle paresis and eventually death, usually due to respiratory failure or pneumonia [1]. In 2009, mutations in the *fused in sarcoma/translocated in sarcoma* (*FUS*) gene were identified as a cause of amyotrophic lateral sclerosis *(FUS-ALS)* [2, 3]. *FUS* mutations resulting in frameshift, nonsense or missense variants affecting the C-terminal region of the protein are typically associated with an aggressive juvenile-onset form of the disease and are frequently identified as de novo. Other missense variants can present with more typical adult-onset ALS with intermediate or even slow progression rates [4–6]. While FUS-ALS commonly presents as spinal ALS, progressive bulbar paresis (PBP) may also be the initial symptom. To date, over 141 *FUS* variants have been reported in ALS patients, although not all have been confirmed as pathogenic [7]. The prevalence of *FUS* variants varies across populations, accounting for approximately 4.0%–6.0% of familial ALS (fALS) cases and 0.7%–1.8% of sporadic ALS cases [8].

A histopathological hallmark of ALS caused by *FUS* mutations is the presence of neuronal cytoplasmic inclusions immunoreactive for FUS [9, 10] . Notably, FUS-ALS patients lack the characteristic phosphorylated TAR DNA-binding protein 43 kDa (pTDP-43) inclusions that are the predominant pathological feature in most other ALS cases [2, 11].

The majority of ALS-associated *FUS* mutations are located in exon 15, which encodes the nuclear localization signal (NLS). These mutations disrupt the NLS, impairing nuclear import and leading to mislocalization of FUS to the cytoplasm [12]. *FUS* variants outside exons 13–15 are rare, and their role in ALS pathogenesis remains uncertain. Although a few such cases have been reported, their pathogenicity is still under debate [13–16]. To date, only a limited number of autopsies have been performed on patients with *FUS* variants outside the NLS region.

Here we investigated three ALS patients carrying different *FUS* variants: two within the NLS domain—R495X and a likely de novo P525L variant—and one outside the NLS, located in the N-terminal region (Q23L). Both patients with NLS variants exhibited prominent cytoplasmic FUS inclusions in motor neurons. In contrast, the Q23L patient lacked FUS inclusions but showed pTDP-43 pathology, suggesting it to be a likely benign variant.

## Results

### Large cytoplasmic inclusions of FUS in a patient carrying the *FUS* R495X variant (patient #1)

**Figure 1:**
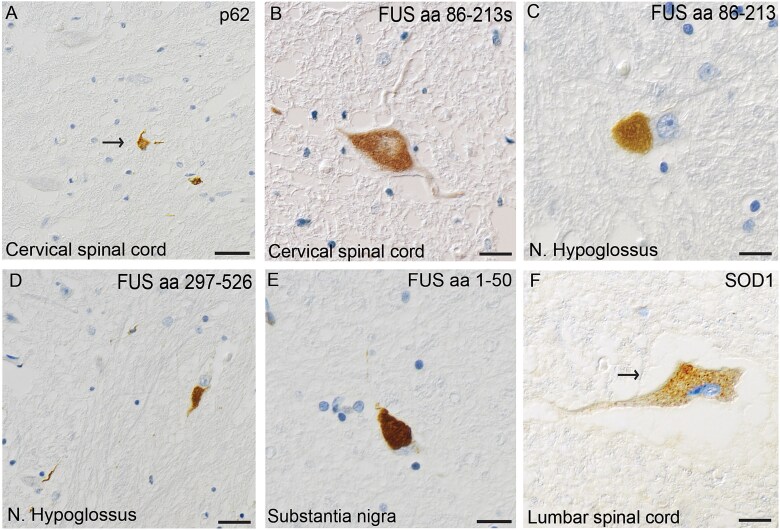
Histological images visualizing inclusions of p62, FUS and SOD1 in patient #1. (A) Depicts p62 cytoplasmic inclusions in ventral horn neurons in the cervical spinal cord. (B) Depicts a large cytoplasmic FUS inclusion in a cervical motor neuron stained with antibody FUS aa 86–213(s). FUS inclusions are also seen in the hypoglossal nucleus stained with the antibody FUS aa 86–213 in (C) and with FUS aa 297–526 in (D) and in substantia nigra with FUS aa 1–50 in (E). (F) Depicts small dot-like SOD1 inclusions, seen with the 131–153 Ra-ab, in a lumbar motor neuron. Scale bars represent 100 μm in A, 50 μm in D and 25 μm in B, C, E and F.

The first patient experienced significant reading difficulties during childhood. She presented at age 16 with dysarthria and dysphagia and was diagnosed with progressive bulbar paresis (PBP) (Table 1, #1). The disease progressed rapidly, leading to severe head drop and involvement of the upper limbs, with signs of both upper and lower motor neuron degeneration. Despite profound weakness—barely able to support her head with her arms—she remained ambulatory until the day before she died of pneumonia, 10 months after symptom onset. Her family history was notable for ALS; her grandmother died of PBP at age 47. Genetic analysis was performed and identified a heterozygous *FUS* R495X variant. Macroscopic findings revealed extensive muscle atrophy of the tongue. Both the pectoralis major and the psoas major muscles showed atrophy but to a lesser extent. Microscopically, hematoxylin–eosin staining revealed loss of motor neurons at all levels of the spinal cord and of the hypoglossal nucleus in the medulla oblongata, as well as cortical Betz neurons. Neuronal cytoplasmic inclusions were visible in the spinal motor neurons, but they were not apparently basophilic. Luxol staining showed pallor with mild loss of myelin in the corticospinal tract and compact cytoplasmic inclusions in spinal motor neurons were seen when staining with an antibody against p62 (Fig. 1A). All four anti-FUS antibodies revealed cytoplasmatic compact, tangle-like and globular as well as a few neuritic inclusions in both the motor cortex and spinal motor neurons, and in the lentiform nucleus, pons, mesencephalon and medulla oblongata (Table 2, Fig. 1B–E). Occasionally ovoid inclusions in glial cells were seen. Staining with the SOD1 131–153 Ra-ab peptide antibody revealed multiple small dot-like SOD1 inclusions in the cytoplasm of motor neurons in both the ventral horn and hypoglossal nuclei (Fig. 1F) as well as in axons and dendrites. Staining for pTDP-43, Tau, β-amyloid and α-synuclein was negative. No signs of small vessel disease, thickening of blood vessel walls, hemosiderin deposits or lacunar infarcts were observed (Table 3).

**Table 1:** Summary of clinical and genetic features.

**Patient**	**1**	**2**	**3**
*FUS* mutation	p.R495X	p.P525L	p.Q23L
	c.1483C > T	c.1574C > T	c.68A > T
*C9orf72HRE* mutation	No	No	No
*SOD1* mutation	No	No	No
Sex	Female	Female	Male
Family medical history	Yes	No	Yes
Age at onset (y)	16	22	35
Age at death (y)	17	23	49
Disease duration (m)	10	12	174
Site of onset	Bulbar	Left arm	Bulbar
Cognitive impairment	No	No	Yes
Early ID	No	Yes	No
Affect lability	Yes	No	Yes
UMN symptoms	Present	Present	Dominating
LMN symptoms	Dominating	Dominating	Present
RIG	Yes	Yes	No
NIV	Yes	No	Yes
Cause of death	Aspiration pneumonia	Pneumonia	Pneumonia
Postmortem time (h)	96	72	72

Abbreviations used: *FUS*, fused in sarcoma; *C9orf72HRE*, C9orf72 hexanucleotide repeat expansion; *SOD1*, superoxide dismutase 1; *y*, years; *m*, months; *early ID*, early intellectual disability; *UMN*, upper motor neuron;

*LMN*, lower motor neuron; *EMG*, electromyography; *RIG*, radiologically inserted gastrostomy; *NIV*, noninvasive ventilation; *h*, hours.

**Table 2:** FUS immunohistochemistry.

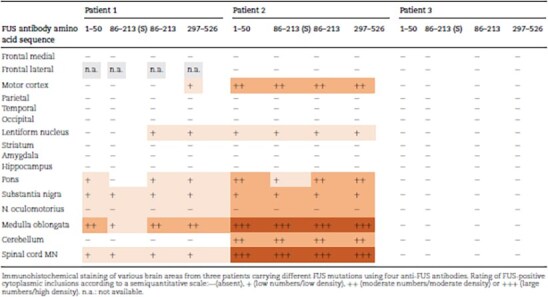

**Table 3:** Summary of various neurodegenerative and vascular pathologies.

**Patient**	**NIA-AA ABC score**	**Tauopathy**	**LBD**	**CAA**	**SVD**
**1**	A0B0C0, not	No	No	No	No
**2**	A0B0C0, not	No	No	No	No
**3**	A0B1C0, not	PART	No	No	Yes

*NIA-AA ABC score:* National Institute on Alzheimer’s Association ABC score. A = A score converted from the Thal beta-amyloid phase, B = B score converted from the Braak and Braak NFT stages, C = CERAD (Consortium to Establish a Registry for Alzheimer’s Disease) neuritic plaque score. *PART*, primary-aging-related tauopathy; LBD, Lewy body disease; CAA, cerebral amyloid angiopathy; SVD, small vessel disease.

### Large globular and elongated cytoplasmic FUS inclusions were present in a patient carrying a possible de novo P525L *FUS* variant (patient #2)

**Figure 2:**
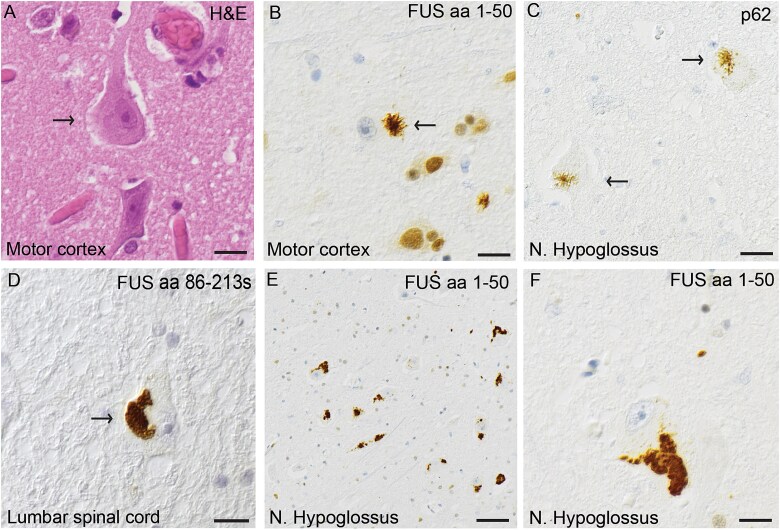
Histological images with basophilic cytoplasmic inclusion and representative inclusions of FUS and p62 in patient #2. (A) Depict a basophilic cytoplasmic inclusion (arrow) in a neuron located in motor cortex layer V. (B) Depicts a large cytoplasmic FUS inclusion in a Betz cell in motor cortex as well as retained nuclear FUS immunoreactivity visualized with anti-FUS aa 1–50. (C) Depicts hypoglossal nuclei motor neurons with p62 inclusions. In (D) a large cytoplasmic FUS inclusion in a lumbar spinal cord motor neuron is depicted, stained with antibody FUS aa 86–213(s). (E) Depicts several hypoglossal nuclei motor neurons with cytoplasmic FUS inclusions visualized with anti-FUS aa 1–50. (F) Depicts a large cytoplasmic FUS inclusion in the hypoglossal nucleus. Scale bars represent 100 μm in E, 50 μm in B, C, and 20 μm in A, D and F.

**Figure 3:**
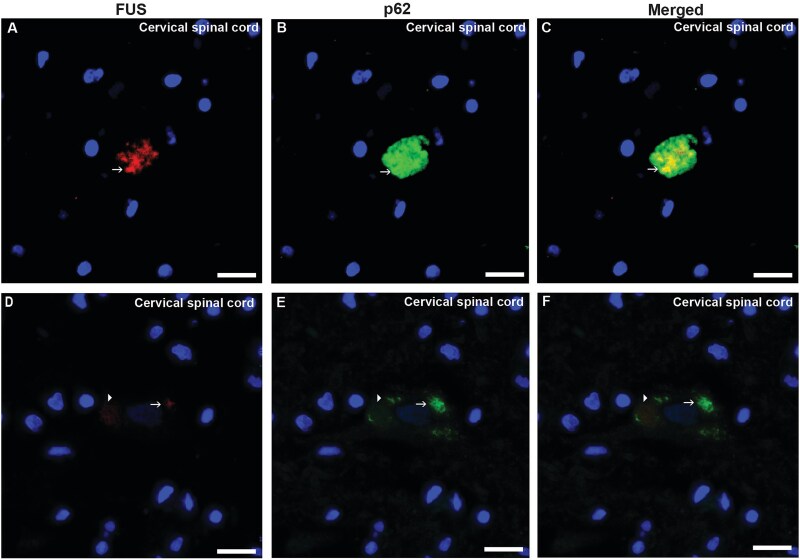
Histological sections from the cervical spinal cord of patient #2 carrying the *FUS* variant P525L. Sections were double-labeled with the anti-FUS (aa 86–213 s) and anti-p62 (aa257–437). Panel (A) shows a cytoplasmic FUS inclusions (red) in a motor neuron and panel (B) shows a large cytoplasmic p62 inclusion in the same motor neuron (green). The merged panel (C) shows that FUS and p62 partly overlap (yellow). Panel (D) shows a small cytoplasmic FUS inclusion (red) in a motor neuron and in panel (E) larger cytoplasmic inclusions of p62 (green) are depicted. The merged panel (F) shows that some of the inclusions are localized to the same area (F, arrow), whereas other inclusions of p62 do not localize to FUS staining (arrowhead). Scale bars represent 25 μm in A–F.

The second patient was a 22-year-old woman who initially developed paresthesia in her left hand following a mild throat infection (Table 1, #2). This was soon followed by visible atrophy in the left upper arm. The disease progressed rapidly, and within weeks, her left arm became flaccidly paralyzed. The condition continued to spread to all four limbs, with predominant lower motor neuron (LMN) signs, although upper motor neuron (UMN) signs were also present. Bulbar involvement was mild and appeared only in the later stages. She died from bronchitis/pneumonia 12 months after symptom onset. Mild intellectual disability had been noted since early childhood, and she had attended special education classes. There was no known family history of motor neuron disease or frontotemporal dementia. Initially diagnosed with sporadic ALS, genetic testing later revealed a heterozygous *FUS* P525L variant. The mutation was absent in her biological mother. Her biological father, who is asymptomatic, declined genetic testing. Macroscopic investigations revealed muscle atrophy in pectoralis major, psoas major, gastrocnemius and tongue, which was confirmed histopathologically. Hematoxylin–eosin staining revealed spinal motor neuron loss and ventral horn gliosis. A few motor neurons had basophilic inclusions (Fig. 2A). The anti-FUS antibody labeled large globular and elongated cytoplasmic inclusions in cervical, thoracic and lumbar spinal cord motor neurons, including dystrophic neurites (Fig. 2B–F). Some retention of nuclear FUS immunoreactivity was observed with anti-FUS aa 1–50 and anti-FUS aa 297–526 antibodies. Double-label immunofluorescence staining with FUS and p62 revealed the colocalization of inclusions in a few neurons, whereas other neurons appeared without merged immunofluorescence staining (Fig. 3). Staining for pTDP-43, Tau, β-amyloid, α-synuclein and SOD1 was negative. No signs of small vessel disease were observed (Table 3).

### No cytoplasmic FUS inclusions were detected in a patient carrying *FUS* Q23L, thereby questioning the pathogenicity of the variant (patient #3)

The third autopsy involved a 35-year-old man who initially presented with a gradual onset of dysarthria and dysphagia (Table 1, #3). Demographic data for this patient has previously been described in Naumann et al. [6]. Five years into the disease, he developed anarthria, affective lability, and later moderate cognitive impairment. The bulbar symptoms were followed by slowly progressive limb involvement, predominantly affecting the left side with upper motor neuron (UMN) signs. Over time, he developed severe generalized spasticity. Despite the UMN predominance, repeated EMG examinations revealed muscle atrophy and neurogenic changes. The patient died of pneumonia 14 years after symptom onset. Genetic analysis identified a heterozygous Q23L variant in *FUS.* This variant was reported in the gnomAD (Genome Aggregation Database) to have a very low allele frequency (0.000002542 in European non-Finnish population) and a REVEL (Rare Exome Variant Ensemble Learner) score was predicted to 0.649. Given the patient’s family history of frontotemporal dementia (FTD) in a maternal uncle, the genetic analysis was extended to include the most common genes associated with FTD (*TARDBP, GRN, TBK1, CHMP2B*, *SQSTM1, UBQLN2, VCP* and *MAPT*) but no additional variants of clinical significance were detected. DNA from the uncle was not available for analysis.

**Figure 4:**
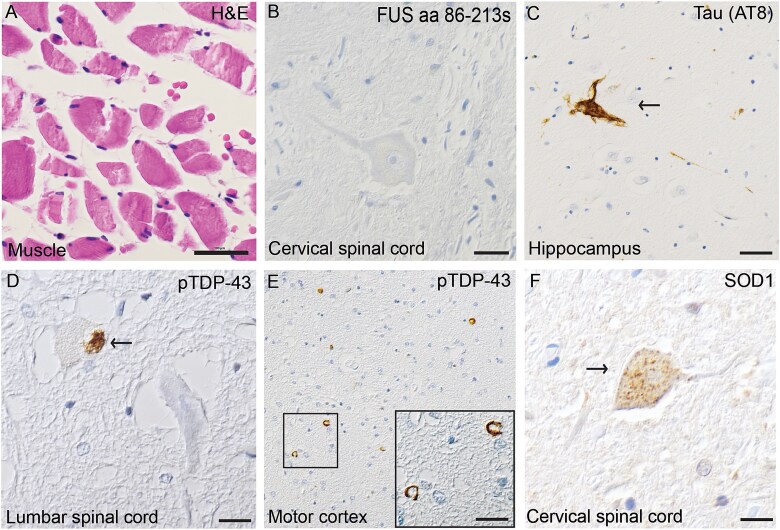
Histological images visualizing muscle atrophy and inclusions of pTDP-43, tau (AT8) and SOD1in patient #3. (A) Depicts muscle atrophy in M. Psoas major. (B) Depicts negatively stained cervical spinal cord motor neuron, anti-FUS aa 86–213(s). (C) Depicts tau(AT8) positive neuronal neurofibrillary tangle in the hippocampus, diagnosed as primary aging-related tauopathy (PART). (D) Depicts phosphorylated TAR DNA-binding protein 43 (pTDP-43) inclusions in lumbar spinal cord motor neurons (arrow) whereas (E) depicts pTDP-43 inclusions in the motor cortex. (F) Small dot-like SOD1 inclusions are seen with the 131–153 Ra-ab in a cervical spinal cord motor neuron. Scale bars represent 100 μm in A, 50 μm in E and 25 μm in B-D, F.

The neuropathological examination revealed muscle atrophy in the psoas major muscle (Fig. 4A) and, to a lesser extent, in the diaphragm. Hematoxylin–eosin staining revealed ventral horn gliosis and spinal motor neuron loss in the cervical and thoracic spinal cord and only mild loss in the lumbar spinal cord. Several motor neurons had a swollen eosinophilic appearance, and no basophilic inclusions were observed. Luxol fast blue staining revealed mild pallor with myelin loss in the corticospinal tract. Some loss of neurons could be observed in the hypoglossal nucleus. Immunohistochemistry with all four anti-FUS antibodies lacked FUS inclusions in all investigated areas (Table 2, Fig. 4B). Staining for ubiquitin and p62 revealed a few cytoplasmic compact inclusions in the spinal motor neurons, motor cortex and inferior olivary nuclei. pTDP-43 staining showed few inclusions in spinal cord motor neurons (Fig. 4D) as well as in the inferior olivary nuclei, lentiform nucleus, striatum, and pons in small amounts. In the frontal, parietal and temporal cortices, as well as in the motor cortex, pTDP-43 compact and crescent shaped inclusions (Fig. 4E) were more frequent and appeared mainly in cortical layer II. A few short dystrophic neurites were also present. The morphology was best suited to subtype B [17, 18], with some characteristics of subtype A since most inclusions were compact, not granular, and in cortical layer II. However, no intranuclear inclusions could be found. The most frequent pTDP-43 inclusions were observed in the hippocampus and amygdala whereas the hypoglossal nuclei and cerebellum were negative. β-amyloid and α-synuclein were negative in all stained areas, including the hippocampus and mesencephalon (Table 4). Tau staining was negative in all stained regions apart from a few positive neurofibrillary tangles in the hippocampus compatible with the neuropathological diagnosis of primary age-related tauopathy (PART) (Table 3, Fig. 4C). The SOD1 131–153 Ra-ab visualized cytoplasmatic small granular inclusions in motor neurons at all levels of the spinal cord (Fig. 4F). No lacunar infarcts were observed, but small vessel disease with thickening of the vessels appeared in the lentiform nucleus. According to the VCING model, the likelihood that small vessel disease contributed to the patient’s cognitive impairment was low [19].

**Table 4:** Immunohistochemical staining of samples from patient 3.

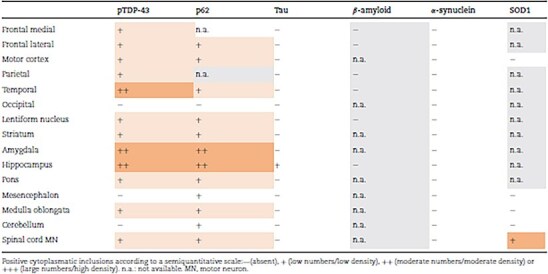

### Control patients

No cytoplasmic FUS inclusions could be detected by any of the four anti-FUS antibodies in the three ALS patients or the other three individuals. In some control patients, weak, diffuse nuclear staining was visible with anti-FUS aa 1–50 and anti-FUS aa 297–526 antibodies (Supplementary Table 3).

## Discussion

Validating the pathogenicity of *FUS* variants using postmortem tissue has become increasingly important with the development of FUS-targeted therapies. Here, we present neuropathological findings from three ALS patients carrying different *FUS* variants. Two patients, with mutations located in the nuclear localization signal (NLS)—R495X and a likely de novo P525L—exhibited aggressive, juvenile-onset ALS with multiple neuronal cytoplasmic FUS inclusions distributed in the brain and spinal cord, supporting previously reported (classical) FUS neuropathology [9, 10] (Fig. 1B–E, Fig. 2B, D–F).

In contrast, the third patient carried the Q23L variant located in the N-terminal region of *FUS*. Immunohistochemistry using four different anti-FUS antibodies was negative across all examined regions (Fig. 4B). Instead, compact and granular pTDP-43 inclusions were observed throughout the brain and brainstem, as well as in a subset of spinal motor neurons (Fig. 4D–E). The large number of pTDP-43 inclusions in the motor cortex may underlie the severe generalized spasticity observed clinically. Additionally, small granular SOD1-positive inclusions were detected in spinal cord motor neurons (Fig. 4F), which may have contributed to downstream pathological processes.

A notable feature of *FUS*-associated ALS is the relatively high frequency of de novo mutations [20–22]. Both the P525L and R495X mutations have previously been reported as arising de novo [23, 24]. In our cohort, Patient #2 carried the P525L mutation and reported no family history of motor neuron disease. Genetic testing of the biological mother was negative for known ALS-causing mutations. Although DNA from the biological father was unavailable, there was no known history of ALS in his family, and he remains asymptomatic in his fifties. Additionally, no other ALS patients with the P525L mutation have been identified in this geographic region. While definitive confirmation is not possible, these findings strongly suggest that the P525L mutation in this patient likely arose de novo.

An association between *FUS* mutations and early-life intellectual disabilities (IDs) has been reported, particularly in cases involving variants that disrupt the NLS [25]. The P525L mutation, in particular, has been linked to a relatively high rate of ID occurrence (19.0%) [4, 25]. In our cohort, Patient #1 exhibited significant learning difficulties, while Patient #2 had a documented mild intellectual disability and attended a special education class during childhood. Neither patient showed tau-, β-amyloid-, nor α-synuclein-positive inclusions, supporting a potential link between these *FUS* mutations and neurodevelopmental features.

Interestingly, Patient #3, who carried the Q23L variant, developed moderate cognitive impairment over the extended course of his illness, with end-stage clinical features consistent with frontotemporal lobar degeneration with TDP-43 pathology (FTLD-TDP). Neuropathological analysis revealed widespread pTDP-43 pathology most consistent with FTLD-TDP type B [17, 18], though the inclusions were fewer and more granular compared to classic type B, and no intranuclear inclusions were observed. Additionally, age-related tau pathology consistent with primary age-related tauopathy (PART) [26] may have contributed to his cognitive decline.

The absence of FUS cytoplasmic inclusions in the Q23L variant raises questions regarding its disease-causing potential. Only a few ALS patients carrying N-terminal *FUS* variants have been described—including S57del, S96del, G156E, G174del, G187S, G191S, and R216C [13–16, 27, 28]. To our knowledge, only one autopsy case involving a patient with an N-terminal variant—FUS p.G174del—has been reported [27]. Similar to our findings, that case lacked cytoplasmic FUS inclusions and instead exhibited pTDP-43 pathology in the spinal cord. Two other N-terminal FUS variants close to the Q23L have been described in the ClinVar database: Y25S and P18S. Both were classified as variants of uncertain significance (VUS). We propose that the Q23L variant is likely benign based on the following: 1) the clinical phenotype, characterized by a slowly progressive disease with predominant upper motor neuron signs, is atypical for pathogenic FUS mutations; 2) postmortem analysis revealed no FUS inclusions in the motor cortex or spinal cord and 3) the presence of pTDP-43 pathology, which is not observed in confirmed cases of FUS-ALS [2] and further supports a different disease mechanism; 4) The genetic region harbouring the mutation shows no regional constraint (Z score − 3.59), in contrast to the regions where clearly pathogenic variants are located.

A criteria that potentially could support a pathogenic role of the variant is the REVEL score, which was just above the threshold described by Pejaver et al [29], but is below the threshold suggested by ACGS [30]. Moreover, a study using multiple human motor neuron cultures—including one derived from the Q23L patient—reported that mutant *FUS* impact the metabolic state [31]. Also, the early age of onset observed in the Q23L patient does not exclude the possibility that the *FUS* variant could act as a potential risk factor.

In summary, our findings support that *FUS* variants within the NLS region lead to mis-localization of the FUS protein to the cytoplasm and result in a toxic ‘gain-of-function’ effect likely contributing to juvenile onset and aggressive disease progression. In contrast, the N-terminal Q23L variant did not result in cytoplasmic FUS aggregation, which questions the pathogenicity of the variant and suggests it as likely benign. Given that antisense oligonucleotide (ASO)-mediated *FUS* silencing is currently under investigation as a therapeutic strategy, neuropathological investigations are crucial for understanding its potential impact in patients with non-NLS mutations. Further research is needed to guide future treatment strategies.

## Materials and methods

### Patients

The patients were diagnosed in accordance with the European Federation of Neurological Sciences (EFNS) guidelines for the clinical management of ALS [32] and the El Escorial criteria [33]. The family history was considered positive for ALS if the patient had at least one affected relative within three generations. Autopsies were performed at Umeå University Hospital, with postmortem times ranging from 72–96 h (Table 1). The study was approved by the Swedish Ethical Review Authority (SERA no. 1994–135, with amendments in 1998, 2003, 2014, 2017 and 2018) for the molecular and DNA analysis and EPN2014-17-31 M for studies on CNS tissue. Written informed consent was obtained from all patients and/or their next of kin. All procedures were performed in accordance with the 1964 Declaration of Helsinki and its subsequent amendments.

### Molecular genetic analysis

DNA was extracted from blood leukocytes using NUCLEON BACC3 (GE Healthcare) according to the manufacturer’s instructions. All patient DNA samples underwent Sanger sequencing of FUS exons 13–15 including at least 30 base pairs (bp) of flanking intronic regions (for primer sequences, see Supplementary Table 1). For Patient #3, comprehensive sequencing of all FUS exons with ≥30 bp of flanking intronic sequences was performed. Additional screening included SOD1 sequencing following established protocols as previously described in [34], and analysis of the *C9ORF72*HRE using fragment length analysis and repeat-primed PCR [35]. Sequencing was conducted on a 3500 Genetic Analyzer (Applied Biosystems) with data analysis via SeqScape v3.0 software (Applied Biosystems). Patient #3 also underwent whole-genome sequencing as part of the Project MinE initiative [36]. Subsequent analyses targeted the following ALS-associated genes: *TARDBP, GRN, TBK1, CHMP2B*, *SQSTM1, UBQLN2, VCP* and *MAPT.* No pathogenic variants were identified, however the data quality for MAPT was suboptimal. Variants interpretation adhered to the American College of Medical Genetics and Genomics (ACMG) guidelines [37].

### Tissue collection, preparation and immunohistochemistry

At autopsy, tissues from the spinal cord (cervical, thoracic and lumbar), medulla oblongata at the midlevel of the inferior olive, pons at the midlevel, mesencephalon at the level of the third cranial nerve, cingulate gyrus, lateral frontal lobe (middle frontal gyrus), parietal lobe (superior or inferior parietal lobule), temporal lobe lateral aspects, posterior hippocampus, occipital lobe (calcarine sulcus), striatum/caudate nucleus at the level of nucleus accumbens, amygdala, lentiform nucleus and cerebellum with dentate nuclei. Additional skeletal muscles included the pectoralis major, the psoas major, the gastrocnemius, the diaphragm, and the hypoglossal muscle. Tissue for immunohistochemical analyses were immersion-fixed in 10% neutral buffered formalin at room temperature, and the samples used for DNA analyses were freshly frozen and stored at −80°C.

Formalin-fixed, paraffin-embedded tissue blocks were sectioned at 4 μm and stained with hematoxylin/eosin and Luxol fast blue according to standard protocols [38]. For immunohistochemistry, staining was performed according to the manufacturer’s recommendations using the BenchMark Ultra (Ventana Medical Systems, Roche Group, Tucson, AZ, USA) with either the UltraView or the OptiView detection kits. The sections were counterstained with hematoxylin, washed and mounted with Glycergel Mounting Medium (DakoCytomation). The following commercially available antibodies were used: FUS (four antibodies with three different epitopes: aa 1–50; aa 86–213(s); aa 86–213; and aa 297–526), p62, pTDP-43 [9–11], hyperphosphorylated Tau (AT8), β-amyloid (6F/3D), α-synuclein (KM51) and ubiquitin (Supplementary Table 2). SOD1 inclusions were stained with a SOD1 peptide antibody against amino acids 131–153, (SOD1 131–153 Ra-ab) as described previously [39]. The presence of intracellular misfolded SOD1 inclusions was rated according to a 4-point semiquantitative scale [39].

Double-label immunofluorescence staining was performed on selected samples for FUS (aa 86–213 (Sigma)) and p62. Alexa Fluor 594- and Alexa Fluor 488-conjugated anti-mouse and anti-rabbit IgG secondary antibodies were used. The sections were mounted with fluorescence mounting medium containing DAPI for nuclear counterstaining. Immunohistochemical and immunofluorescence images were obtained using a fluorescence microscope (Nikon Eclipse Ni-L) and processed via the software Nikon NIS-Elements BR5.42.0464-bit.

FUS, p62, pTDP-43 and α-synuclein levels were evaluated on a semiquantitative scale of 0 (absent), 1 (small numbers/low density), 2 (moderate numbers/moderate density) or 3 (large numbers/high density). At least 4 sections per brain region were evaluated for FUS and 2 sections per brain region for pTDP-43. Examples of FUS grading can be seen in Supplementary Fig. 1. Alzheimer’s disease staging was performed using the NIA-AA ABC score [40]. Small vessel disease and the presence of lacunar infarcts were evaluated [19]. The sections were examined using a Nikon Eclipse Ci light microscope by two neuropathologists (ES and ZR) who were blinded to the genotypes of the patients.

### Controls

Comparisons were made with CNS tissue from three ALS patients lacking *FUS* mutations and three control individuals without neurodegenerative disease (Supplementary Table 3). The ALS patients consisted of two patients with SALS and one patient carrying a *C9orf72HRE* mutation. Tissue sampling, sectioning and staining were performed as described above.

## Supplementary Material

Supplementary_Material_HmG_ddaf119
