# Supplementary material for: Pathology of three ALS patients with FUS variants, including one likely benign Q23L variant lacking FUS inclusions
Source: Hum Mol Genet. 2025 Jul 15;34(18):1553–62. doi: 10.1093/hmg/ddaf119 (PMC12409623; doi:10.1093/hmg/ddaf119)
Supplement: Supplementary_Material_HmG_ddaf119 [file supplementary_material_hmg_ddaf119.docx]

Supplementary Table 1. The FUS primer sequences (5'-3')

| **FUS exon** | **Primer sequence 5'-3'** |
| --- | --- |
| Exon 1F | ttcatgggggtggagataga |
| Exon 1R | ctgacgcttcaaacccctaa |
| Exon 2-3F | ctcaagtgatccacccacct |
| Exon 2-3R | aggcaggagaatcgtttgaa |
| Exon 4F | gtttagagggtggtgctgga |
| Exon 4R | acacaatcacaatcccagca |
| Exon 5F | gtacggtggtgttgatgtcg |
| Exon 5R | tcaggagatcgaaaccatcc |
| Exon 6F | ggatggtttcgatctcctga |
| Exon 6R | aggctcccaagttctcacaa |
| Exon 7F | atctgctccatcggaagaac |
| Exon 7R | ccacacctggcacttagtga |
| Exon 8F | gccaagctgagttggtttgt |
| Exon 8R | ctaaatcactgggggtggaa |
| Exon 9F | tcagcatggctggcatatag |
| Exon 9R | aaggctctttcctcaacaaat |
| Exon 10F | ggaggtttacatgtgaggtagg |
| Exon 10R | cattacccctctgcaccact |
| Exon 11F | gaggcaacggtctcttttga |
| Exon 11R | ctccactgggaaatcctcct |
| Exon 12F | agactttaatcggggtggtg |
| Exon 12R | gcttgcttctcagagtcagga |
| Exon 13-15F | gactctgagaagcaagccgt |
| Exon 13-15Ra | cgggacatcgatcttccagg |
| Exon 13-15Rb | ccttgggtgatcaggaattg |
| Exon 13-14R | atggcctctgttcaactgct |

Supplementary Table 2. Commercial and in-house antibodies

| **Description** | **Product identification** | **Clone** | **Amino acid sequence** | **Manufacturer** | **Clonality** | **Antibody dilution** |
| --- | --- | --- | --- | --- | --- | --- |
| Rabbit anti-FUS | MA5-44241 | BLR023E | 1-50 | Invitrogen | Monoclonal | 1:100 |
| Rabbit anti-FUS | HPA008784 | - | 86-213 | Sigma | Polyclonal | 1:1000 |
| Rabbit anti-FUS | PA5-52610 | - | 86-213 | Invitrogen | Polyclonal | 1:1000 |
| Rabbit anti-FUS | PA5-96477 | - | 297-526 | Invitrogen | Polyclonal | 1:100 |
| Mouse anti-p62 | 610833 | - | 257-437 | BD Transduction Laboratories | Monoclonal | 1:50 |
| Mouse anti-TDP-43 | CAC-TIP-PTD-M01A | 11-9 |  | Cosmo Bio LTD | Monoclonal | 1:3000 |
| Mouse anti-human  β-amyloid | M087201-2 | 6F/3D |  | Agilent Technologies | Monoclonal | 1:100 |
| Mouse anti-TAU | BR-03 | AT8 |  | Innogenetics | Monoclonal | 1:800 |
| Mouse anti-α-synuclein | NCL-L-ASYN | KM51 |  | Novocastra | Monoclonal | 1:100 |
| Rabbit anti-ubiquitin | Z0458 | - |  | Dako | Polyclonal | 1:500 |
| Rabbit anti-SOD1 | - | Ikaros | 131-153 | In-house | Polyclonal | 1:1000 |

For the FUS antibodies, heat-induced antigen retrieval with the Ultra CC2 mild program were used for 44 minutes. Primary antibody incubation time were 32 minutes and detected by the the UltraView DAB Detection kit.

Supplementary Table 3. Characteristics of the control patients and results of FUS immunohistochemical staining

| **Patient** | **Diagnose** | **Cause of death** | **FUS**  **(aa 1–50)** | **FUS**  **(aa 86–213(S))** | **FUS**  **(aa 86–213)** | **FUS**  **(aa 297–526)** |
| --- | --- | --- | --- | --- | --- | --- |
| **1** | sALS with pTDP-43 inclusions. Also Lewy body disease in the neocortical stage and advanced Alzheimer’s disease. | Respiratory insufficiency | Focal weak nuclear staining | - | - | Focal weak nuclear staining |
| **2** | fALS with pTDP-43 inclusions. The C9orf72 mutation was verified. | Respiratory insufficiency | Focal weak nuclear staining | - | - | Focal weak nuclear staining |
| **3** | ALS with pTDP-43 inclusions. | Pneumonia | - | - | - | - |
| **4** | Schizophrenia | Acute intracerebral and subarachnoid hematoma | - | - | - | Focal weak nuclear staining |
| **5** | Vasculitis | Acute pontine infarction | - | - | - | Focal weak nuclear staining |
| **6** | Ulcus ventriculi | Myocardial infarction | - | - | - | - |

FUS-positive cytoplasmic inclusions or positive nuclear staining was rated according to a semiquantitative scale: (absent). Focal weak nuclear staining was diffuse with no compact or granular appearance.


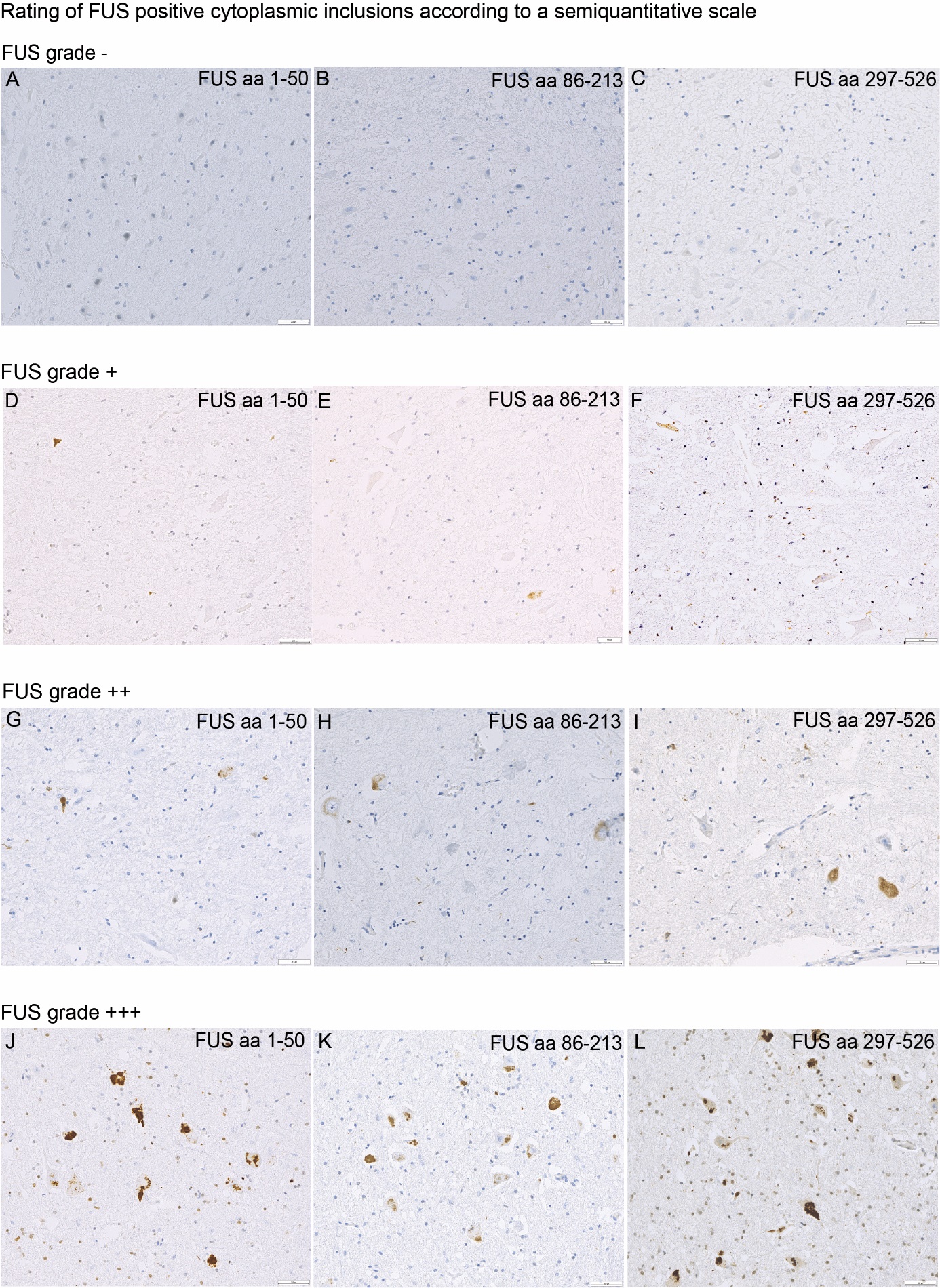
Supplementary Figure 1.

A-C. Patient 3. Hypoglossal nuclei. Motor neurons negative for cytoplasmic FUS inclusions.

D-F. Patient 1. Spinal cord motor neurons with low numbers/density of cytoplasmic positive FUS inclusions.

G-I. Patient 1. Hypoglossal nuclei. Motor neurons with moderate numbers/density of cytoplasmic FUS inclusions.

J-L. Patient 2. Hypoglossal nuclei. Motor neurons with large numbers/density of cytoplasmic FUS inclusions.
